# First Whole-Genome Sequence of a Clinical Isolate of Multidrug-Resistant *Mycobacterium bovis* BCG

**DOI:** 10.1128/genomeA.00611-14

**Published:** 2014-07-10

**Authors:** A. Renvoisé, S. Pang, C. Bernard, F. Brossier, N. Veziris, E. Capton, V. Jarlier, W. Sougakoff

**Affiliations:** a Sorbonne Universités, UPMC Université Paris 06, CR7, Centre d’Immunologie et des Maladies Infectieuses, CIMI, Team E13 (Bacteriology), Paris, France; b INSERM, U1135, Centre d’Immunologie et des Maladies Infectieuses, CIMI, Team E13 (Bacteriology), Paris, France; c AP-HP, Hôpital Pitié-Salpêtrière, Centre National de Référence des Mycobactéries et de la Résistance des Mycobactéries aux Antituberculeux, Bactériologie-Hygiène, Paris, France

## Abstract

The attenuated BCG strain of *Mycobacterium bovis* is widely used as a vaccine against tuberculosis. However, in rare cases, it can be pathogenic to humans. Here, we report the first draft of a whole-genome sequence of a multidrug-resistant clinical isolate of *M. bovis* BCG.

## GENOME ANNOUNCEMENT

Since 1921, bacillus Calmette-Guérin (BCG), an attenuated derivative of *Mycobacterium bovis*, has been used to immunize against tuberculosis, as it is currently the only available vaccine. As of 1974, it has been part of the WHO’s Expanded Program on Immunization because of its proven efficacy in preventing extrapulmonary tuberculosis in children. However, in adults, its efficacy against pulmonary disease varies, possibly due to the use of different derived daughter strains (1–3).

The attenuated strain BCG was derived by Albert Calmette and Camille Guérin at the Institut Pasteur from an *M. bovis* isolate after 230 serial passages between 1908 and 1921 on potato slices soaked with glycerol. Once the safety of the vaccine was confirmed, BCG was disseminated around 1924 to different laboratories that maintained their own daughter strains through passaging, until the introduction of archival seed lots in the 1960s. Consequently, the original strain of BCG has produced many heterogenic daughter strains (substrains), which are the progenitors of the commonly used vaccines (1–6).

After the availability of the complete genomic sequences of *Mycobacterium tuberculosis* (7) and *M. bovis* (8), studies of comparative genomics uncovered regions of difference, such as deletions, insertions, and single nucleotide polymorphisms (SNPs), between the BCG strains (1, 5, 9, 10). A molecular genealogy of BCG based on genomic deletions and duplications has been established (1, 9, 10). Thus far, complete genome sequences have been determined for BCG strains Pasteur (1), Tokyo (5), Moreau (4), Korea (11), and Mexico (9). Draft sequences have been reported for BCG strains China, Danish, Russia, and Tice (6) and Moreau, Frappier, Glaxo, Phipps, Prague, and Sweden (10).

Here, we report the first draft genome sequence of a multidrug-resistant (MDR) clinical isolate of *M. bovis* BCG, BCG-MDR, which was isolated from a patient sputum sample and was found to be phenotypically resistant to rifampin, isoniazid, ethionamide, *para*-aminosalicylic acid, and cycloserine. Very few BCG strains resistant to rifampin or to both rifampin and isoniazid have been identified so far, and none have been sequenced (12–15). The genome sequence was obtained using Illumina sequencing (MiSeq technology), producing a total of 2,894,644 paired-end reads. These sequences were assembled using SPAdes version 3.0.0 (16), resulting in 62 contigs. The draft genome sequence is 4,333,148 bp in length, with a G+C content of 65% and an average coverage of 133×. The genome sequence was annotated using the NCBI Prokaryotic Genomes Automatic Annotation Pipeline (http://www.ncbi.nlm.nih.gov/genomes/static/Pipeline.html). Using this automated pipeline, we identified 3,943 predicted coding sequences; out of them, 3,825 had a functional assignment. It also predicted 65 pseudogenes, 3 rRNAs, and 45 tRNAs.

Comparison with available *M. bovis* BCG genomes indicates that the clinical isolate is related to BCG Tokyo and BCG Russia. Nonsynonymous mutations were identified in genes known to be involved in the development of resistant forms of the *M. tuberculosis* complex. The availability of this genome will allow comparative genomic analysis with other *M. bovis* BCG daughter strains and might provide insights into the emergence of MDR clinical strains of *M. bovis* BCG.

### Nucleotide sequence accession numbers.

This whole-genome shotgun project has been deposited at DDBJ/EMBL/GenBank under the accession no. JNAF00000000. The version described in this paper is version JNAF01000000.
